# Genome-wide prediction of three important traits in bread wheat

**DOI:** 10.1007/s11032-014-0143-y

**Published:** 2014-07-16

**Authors:** Gilles Charmet, Eric Storlie, François Xavier Oury, Valérie Laurent, Denis Beghin, Laetitia Chevarin, Annie Lapierre, Marie Reine Perretant, Bernard Rolland, Emmanuel Heumez, Laure Duchalais, Ellen Goudemand, Jacques Bordes, Olivier Robert

**Affiliations:** 1 UMR GDEC, INRA-Université Clermont II, 5 chemin de Beaulieu, 63039 Clermont-Ferrand Cedex, France; 2 Bioplante-Florimond Desprez, BP41, 59242 Cappelle en Pévèle, France; 3 Bioplante-R2n, 60 rue Léon Beauchamp, 59930 La Chapelle d’Armentières, France; 4 INRA-APBV, Domaine de la Motte, BP 35327, 35653 Le Rheu Cedex, France; 5 INRA UE Lille, 2 chaussée Brunehaut, Estrées-Mons, BP 50136, 80203 Peronne Cedex, France; 6 Colorado State University, Fort Collins, CO 80523 USA

**Keywords:** Genomic selection, *Triticum aestivum* L., Ridge regression, Bayesian LASSO, Random Forest regression, Plant breeding

## Abstract

Five genomic prediction models were applied to three wheat agronomic traits—grain yield, heading date and grain test weight—in three breeding populations, each comprising about 350 doubled haploid or recombinant inbred lines evaluated in three locations during a 3-year period. The prediction accuracy, measured as the correlation between genomic estimated breeding value and observed trait, was in the range of previously published values for yield (*r* = 0.2–0.5), a trait with relatively low heritability. Accuracies for heading date and test weight, with relatively high heritabilities, were about 0.70. There was no improvement of prediction accuracy when two or three breeding populations were merged into one for a larger training set (e.g., for yield *r* ranged between 0.11 and 0.40 in the respective populations and between 0.18 and 0.35 in the merged populations). Cross-population prediction, when one population was used as the training population set and another population was used as the validation set, resulted in no prediction accuracy. This lack of cross-population prediction accuracy cannot be explained by a lower level of relatedness between populations, as measured by a shared SNP similarity, since it was only slightly lower between than within populations. Simulation studies confirm that cross-prediction accuracy decreases as the proportion of shared QTLs decreases, which can be expected from a higher level of QTL × environment interactions.

**Electronic supplementary material:**

The online version of this article (doi:10.1007/s11032-014-0143-y) contains supplementary material, which is available to authorized users.

## Introduction

With the advance in genomics, particularly with next-generation sequencing tools, it has become possible to generate a large number of molecular markers spanning a genome. These genome-wide markers have been used for genomic selection (GS) and genome-wide association studies (GWAS) for qualitative or quantitative traits. A condition for success is that a sufficient level of linkage disequilibrium (LD) exists between adjacent markers and QTLs.

In their pioneering work, Lande and Thompson (1990) introduced a theory for optimization of weights given to each marker associated with a QTL, and they demonstrated that this index was as efficient for the genetic improvement of a population as the phenotypic score. This marker-assisted selection (MAS) approach used only markers which had been previously associated with a QTL. The efficiency of MAS versus phenotypic selection is optimized when the trait has a low heritability, the population size is larger and the QTLs explain a large proportion of the trait variation. Subsequent studies have shown that efficiency is improved when more QTLs with small effects are included (Bernardo et al. 2006; Moreau et al. 1998). Hospital et al. (1997) showed that the use of the marker index would facilitate early selection, bypassing a trait evaluation step and shortening selection cycles, and thus, genetic gain per cycle would increase. MAS has been used for quantitative traits (e.g., Eathington et al. 2007; Blanc et al. 2008) and is currently routinely used by most plant breeding programs. However, the efficiency of MAS can be limited by the first step of QTL identification, when the statistical power has been low for QTL with small effects in smaller population. For complex traits, like grain yield, the most likely hypothesis is that they are controlled by a large number of QTLs with effects below the detection threshold. Therefore, several QTLs are not accounted for by the markers included in the selection index.

A subsequent step was proposed by Whittaker et al. (2000), who proposed including all markers in the selection index and bypassing the QTL identification step. As the number of markers was generally larger than the number of genotypes, classical fixed effect regression models gave inaccurate estimates. Therefore, Whittaker et al. (2000) suggested using ridge regression models to overcome this over-parameterization problem. This approach, GS, was first developed by animal geneticists (Meuwissen et al. 2001) who applied ridge and Bayesian regression models to animal populations for predicting breeding values. The breeding values are calculated from marker effects estimated from the genotypes and phenotypes of a training population. The marker effects are used to calculate breeding values for the target population with only genotypic data, and selections are based on these estimates. This method has been used successfully for dairy cow breeding (Goddard and Hayes 2007). However, as the LD between markers and QTL and the relatedness between samples are reduced in cows from one generation to the next, genomic estimated breeding value (GEBV) predictions will be less accurate (Habier et al. 2007). Therefore, new phenotypic measurements may be needed to re-estimate marker effects in some species (Heffner et al. 2010).

The most efficient use of GS is to replace costly and time-consuming phenotyping with a prediction of the genetic value of the trait under selection. Thus, the main advantages may be cost and selection cycle reduction. To benefit from shorter cycles, the genetic gain per cycle should be close to the expected gain from phenotypic or combined MAS and phenotypic selection. The relative efficiency relies on the accuracy of predicting the observed genetic value from the marker genotype. The accuracy of a prediction is measured by the correlation between the predicted and observed values, and this accuracy relies on the level of LD between a QTL and linked marker. The relevant parameter is the LD level, *r* ^2^, which has demonstrated that the sample size required to detect a QTL by a nearby marker is 1/*r* ^2^ times the population size required for testing and validating the putative QTL (Balding et al. 2007). The accuracy of breeding value predictions will depend on the trait variation captured by the markers. Marker density should optimize LD between markers and QTLs. The extent of LD has been investigated in several animal and plant species and populations within some species. The LD range is expected to be high in biparental populations (Lorenzana and Bernardo 2009) and higher in more complex mating schemes (Blanc et al. 2008; Bernardo and Yu 2007; Heffner et al. 2009; Jannink et al. 2010; Iwata and Jannink 2011; Lorenz et al. 2011). The LD pattern may change from one generation to the next, since recombination reduces the range of LD in heterozygotes and varies between germplasm sources.

The objective of this investigation was to estimate the reliability of GEBV predictions for three agronomic traits—yield, heading date and test weight—when using different training and target populations and the effect of merging different breeding populations to increase the size of the training panel.

## Materials and methods

### Plant materials

Three populations each composed of advanced lines from the breeding programs of the wheat breeders of French National Institute for Agricultural Research (INRA) and plant Biotechnology Company Bioplante were included in this investigation (Bordes et al. 2014). These lines were derived from crosses of the best yielding breeding lines and the most widely grown cultivars.

Two doubled haploid populations, DH1 and DH2 (369 and 344 lines, respectively), and one recombinant inbred population, RIL (341 lines), were investigated at three locations during 3 years—2009 (DH1), 2010 (RIL) and 2011 (DH2). DH1 and DH2 were developed by Bioplante with the maize pollination method from 80 F1 progeny from 117 parents, including 66 recently developed cultivars. Eight to ten lines were randomly drawn from each of the 80 F1-derived DHs. Recombinant inbred lines were developed by INRA between 2000 and 2010 from 55 crosses using 87 parents, including 52 recently developed cultivars. The RILs have undergone 7 to 9 generations of selfing, leading to nearly two times the number of recombinations expected for DH lines. A smaller set of 38 F8 recent INRA lines (RIL2) were also used for validation in 2011. It is worth noticing that these populations do not represent different selection cycles, but rather independent samples, although issued from adapted west Europe germplasm.

### Phenotypic evaluation

Each population was grown at three different locations in France. In 2009, DH1 was grown at Clermont-Ferrand (45.46N, 3.04E), Cappelle-en-Pévèle (50.30N, 3.10E) and Milly-la-Forêt (48.24N, 2.28E). In 2010, RIL was grown at Clermont-Ferrand, Cappelle-en-Pévèle and Estrées-Mons (49.53N, 3.00E). In 2011, DH2 was grown at Cappelle-en-Pévèle, Milly-la-Forêt and Rennes (48.06N, 1.40E). The crop management corresponded to the usual farming practices used at each location for high-yield objectives, which included a dense planting and applications of high levels of N fertilizer (HN) and pesticides. All breeding lines were grown once in 10 m^2^ plots. To take into account possible heterogeneity of the soil, the lines were randomized into 10 sub-blocks that each included four control cultivars. The experimental design included three locations per year and approximately 400 plots per location. The number of genotypes was prioritized over the number of repetitions.

Test weight (TW, kg h L^−1^), grain yield (GY, t ha^−1^ at 0 % humidity) and heading date (HD, days from the January 1) were determined for each plot. To control for intra-block heterogeneity, trait values were adjusted relative to the four control cultivars repeated in each sub-block using the glm and ls means procedures in Statistical Analysis Software (SAS Institute Inc 1999) in the following model:

$$Y_{ij} = \mu + G_{i} + B_{j} + e_{ij}$$

where *Y*_*ij*_ represents the value of the trait under investigation for genotype *i* in sub-block *j*, *µ* represents the general mean, *G* represents the fixed genotypic effect, *B* represents the fixed sub-block effect, and *e*_*ij*_ represents the error term of the model.

Analysis of variance was carried out for each trait using the subplot adjusted values with the following model:

$$Y_{il} = \mu + G_{i} + {\text{Location}}_{l} + e_{il}$$

where *Y*_*il*_ represents the value of the trait under investigation for genotype *i* at location *l*, *G* represents the random genotypic effect, *Location* represents the fixed location effect, and *e*_*il*_ represents the error term of the model.

### Genetic markers

The populations were genotyped with 3,299 DArT markers generated by Triticarte Pty, Ltd. (Canberra, Australia; http://www.triticarte.com.au), including 2,545, 1,572 and 2,236 polymorphic markers for DH1, DH2 and RIL, respectively (1,156 markers were common to the three populations). The genetic map was built from the data of Triticarte for about 60 % of the markers; the others were placed close to the DArT markers where the LD was the highest. LD was calculated as *r* ^2^ with a R application (R Development Core Team 2011). Out of the 3,299 markers, 2,772 have been successfully mapped (Bordes et al. 2013) on the whole genome. Markers were not selected based on minor allele frequency.

### GEBV estimation

Five statistical methods were used to estimate GEBV using DArT markers:

- GBLUP assumes pedigree relationships in the training population, based on marker genotypes, and then estimates breeding values using a BLUP animal model (Henderson 1975). Computations were carried out using the pedigree package in R (Coster 2010).
- Bayesian ridge regression (BRR) uses a Gaussian prior distribution with a variance common to each marker effect (de los Campos and Pérez 2010; Pérez et al. 2010). The prior residual variance and degree of freedom were *S*_*ε*_ = 4.5 and *df*_*ε*_ = 3, respectively, and the prior variance and degree of freedom of marker effects were *S*_*βR*_ = 0.009 and *df*_*βR*_ = 3, respectively. Estimates of lambda were based on a heritability, *h* ^2^ = 0.37. The number of iterations used as burn-in was 20,000, and the number of iterations made in the Gibbs sampler was 60,000. Computations were carried out using the BLR package in R (de los Campos and Pérez 2010).
- Bayesian LASSO uses a Gaussian prior distribution with a marker-specific prior variance for a differential shrinkage of each marker effect (de los Campos and Pérez 2010; Pérez et al. 2010). The prior residual variance and degree of freedom were *S*_*ε*_ = 4.5 and *df*_*ε*_ = 3, respectively, and the prior variance and degree of freedom of marker effects were *S*_*βL*_ = 0.009 and *df*_*βR*_ = 3, respectively. Computations were carried out using the BLR package in R (de los Campos and Pérez 2010).
- Reproducing kernel Hilbert space (RKHS) is a kernel-based method, which relies on a regularization network, support vector regression and support vector classification. It was implemented in R.
- Random Forest is an ensemble classifier that consists of many decision trees and outputs the class that is the mode of the class’s output by individual trees. The method combines Breiman’s “bagging” idea (Breiman 2001) and the random selection of features, introduced by Amit and Geman (1997) in order to construct a collection of decision trees with controlled variation. The randomForest package was used (Breiman and Cutler 2013).

### Accuracy and validation

Since the true breeding value was unknown, genomic prediction accuracies were measured by the Pearson correlation between GEBVs and the observed phenotypic values. Cross-validation methods were as follows:

- Standard single-population cross-validations used one breeding population and randomly sampled 80 % of the genotypes for the training population to estimate marker effects for GEBV of the remaining 20 % genotypes used as the “validation set.” The resampling process was iterated 200 times to estimate an empirical mean and standard deviation using R-language.
- Multi-population cross-validations used a composite breeding population including DH1 + DH2, DH1 + RIL, DH2 + RIL or DH1 + DH2 + RIL
- Cross-population cross-validations used one or two breeding populations to predict GEBV of another population. For example, DH1 was used to predict DH2, DH1 was used to predict RIL, DH2 was used to predict RIL and RIL to predict RIL2.

### Simulations

One hundred QTLs with normally distributed additive effects were generated for a sample of markers common to the training and validation populations. Subsets comprising 10–100 % (in increments of 10) of markers were drawn independently from the common sample to generate the quantitative trait in the training and validation populations. This accounted for variable proportions of QTLs expressed according to interactions. A normally distributed noise was added to generate the desired heritability of the simulated trait.

### Estimate of kinship

Kinship coefficients among the breeding lines were estimated using the Kinship function of the TASSEL software (http://www.maizegenetics.net/tassel/). The coefficients were divided by the average value of the diagonal, 2.34, in order to obtain a value ranging between 0 and 1 for the estimate of coefficient of coancestries.

## Results

### Phenotypic evaluation

Analysis of variance of the three populations DH1, DH2 and RIL indicated a significant genotypic effect (*P* value <0.001) for the three traits—grain yield, test weight and heading date (data not shown). For all traits, a wide range of phenotypic variation was observed for the three populations (Suppl. Table 1). Mean ranges for GY (8.61–10.78 t ha^−1^) and TW (71.2–80.0 kg h L^−1^) were expectedly high for elite breeding lines, and heritabilities higher for HD (0.88), TW (0.82) and lower for GY (0.70).

### Genomic predictions using a single population

**Fig. 1:**
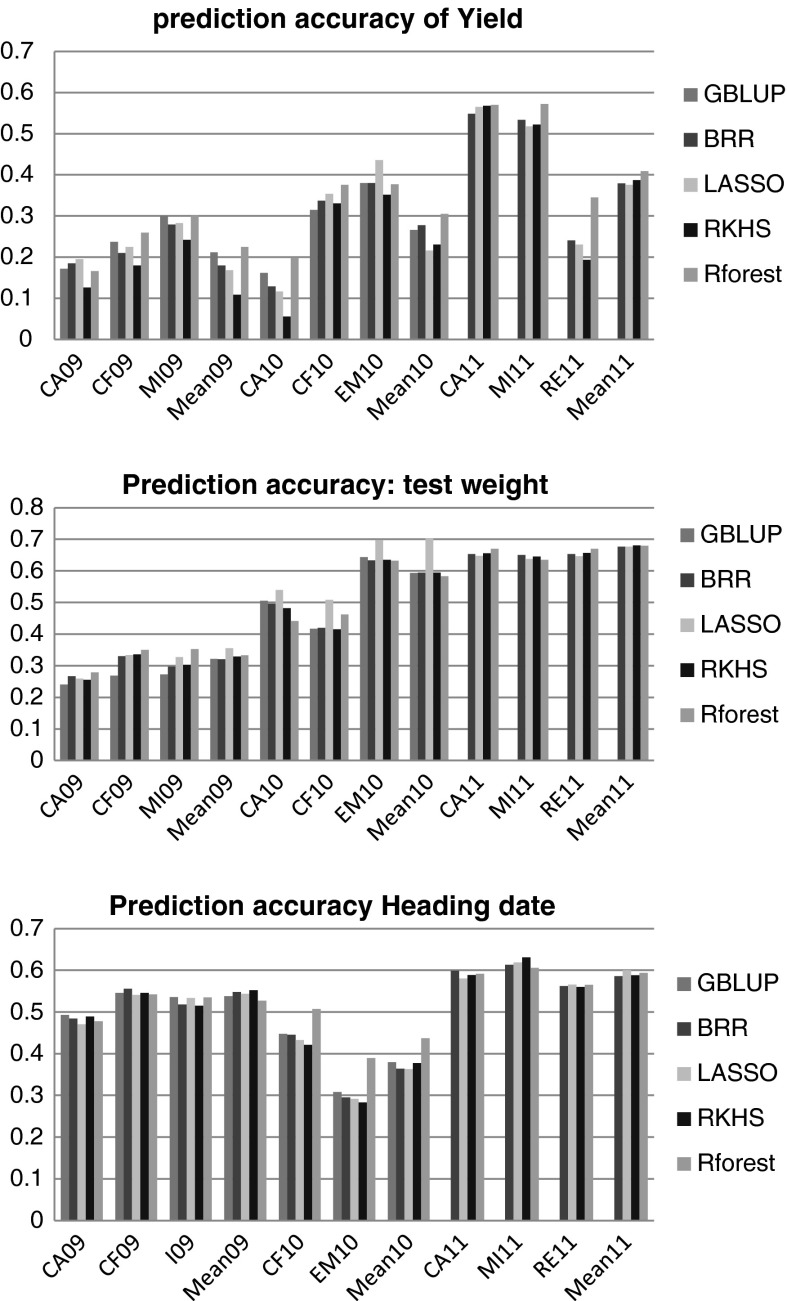
Mean correlations (from 200 resamplings) between the observed trait and GEBV from fivefold cross-validations within a given population (note that GBLUP did not run on the 2011 population, likely due to excessive relatedness between some lines

**Fig. 2:**
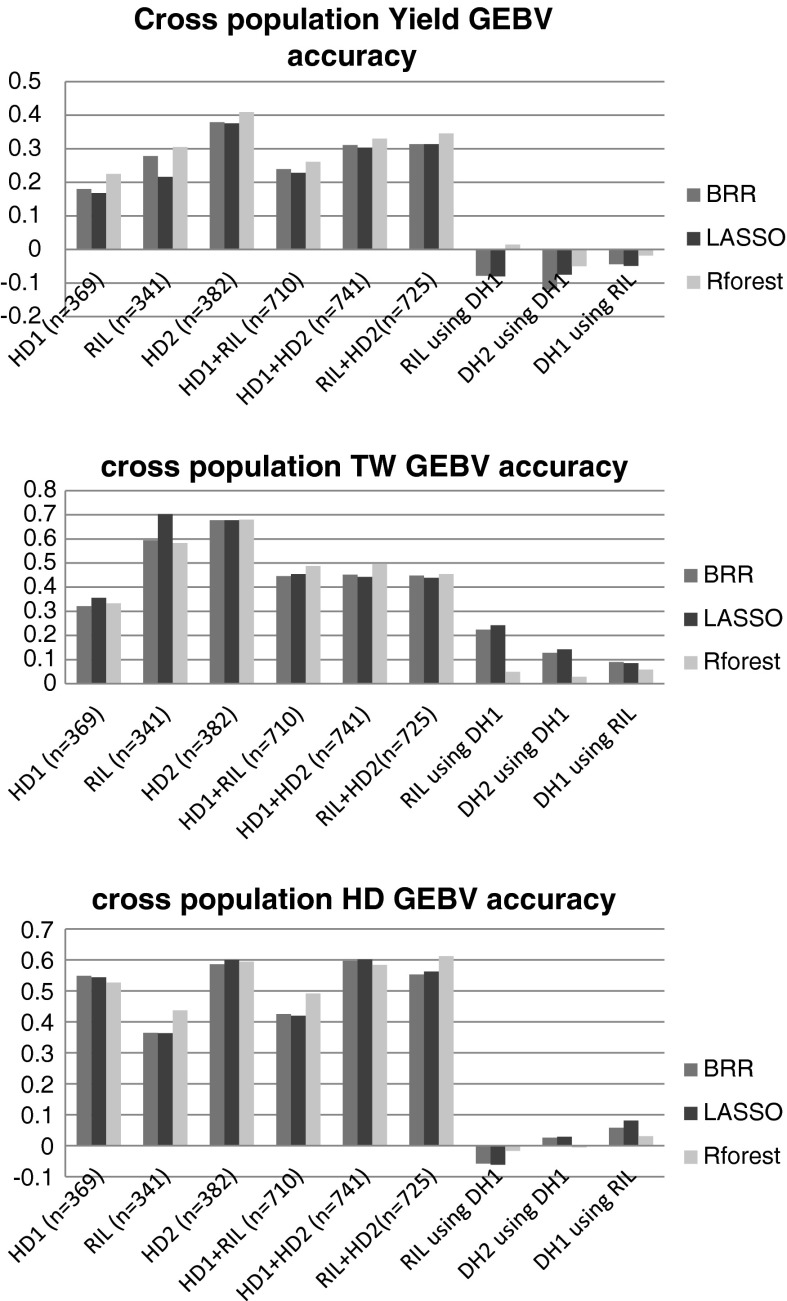
Mean correlations (from 200 resamplings) between the observed trait and GEBV from: (1) single-population cross-validations (*columns 1–3*), (2) composite populations CV (*columns 4–6*) and cross-populations CV (*columns 7–10*). Note that GBLUP did not run on the 2011 population, likely due to excessive relatedness between some lines

 Within-year prediction accuracies are presented in Suppl. Table 2 and Fig. 1. Accuracies vary from one trait to another and one site to another. For GY, they are highly variable, both between years and between locations, with higher average values in 2011 (*r* = 0.216–0.305), with the DH2 populations. For TW, accuracies were more variable between years than between locations, with higher values in 2010 (*r* = 0.583–0.702) and 2011 (*r* = 0.677–0.681) compared to 2009 (*r* = 0.321–0.356). DH accuracy was more stable between locations. The statistical models, LASSO and/or Random Forest, facilitated higher accuracies than other models in some of the comparisons (Figs. 1, 2).

### Genomic predictions using a multi-populations cross-validation

Standard cross-validation accuracies from randomly sampling the training and validation sets obtained on composite populations are presented in Suppl. Table 3. The range of accuracies for single populations (*n* = 341–382) was *r* = 0.109–0.409 and for three merged populations (*n* = 1,092) was *r* = 0.238–0.312. These results suggest that prediction accuracies did not improve with increased training population sizes, when unrelated populations from different breeding programs were merged to increase the population. Instead, the best accuracies estimated for yield in DH2 for 2011, *r* = 0.376–0.409 were somewhat reduced when DH2 merged with any of the other populations (*r* = 0.238–0.346). For HD, we observed more consistent results between admixed populations, which was the case for the single-population cross-validation (*n* = 341–382, *r* = 0.363–0.600), but there were no accuracy improvements from an increased training population size (*n* = 1,092, *r* = 0.488–0.561.

### Genomic predictions using different populations as training and validation sets

Suppl. Table 4 shows the average correlations between GEBV and the observed trait when one population was used for sampling 80 % of its lines for the training set and another population was used for sampling 20 % of its lines for the validation set. Accuracies for yield ranged between −0.12 and 0.015. Figure 2 summarizes the comparison of accuracies between the three cross-validation methods—single, composite and cross-populations. Results indicate that GEBV estimated from one population did not predict phenotypes in a different population. Low accuracies were estimated for all three traits (*r* ranged between −0.12 and 0.24).

### Genomic predictions using cross-population validation based on simulated traits

**Fig. 3:**
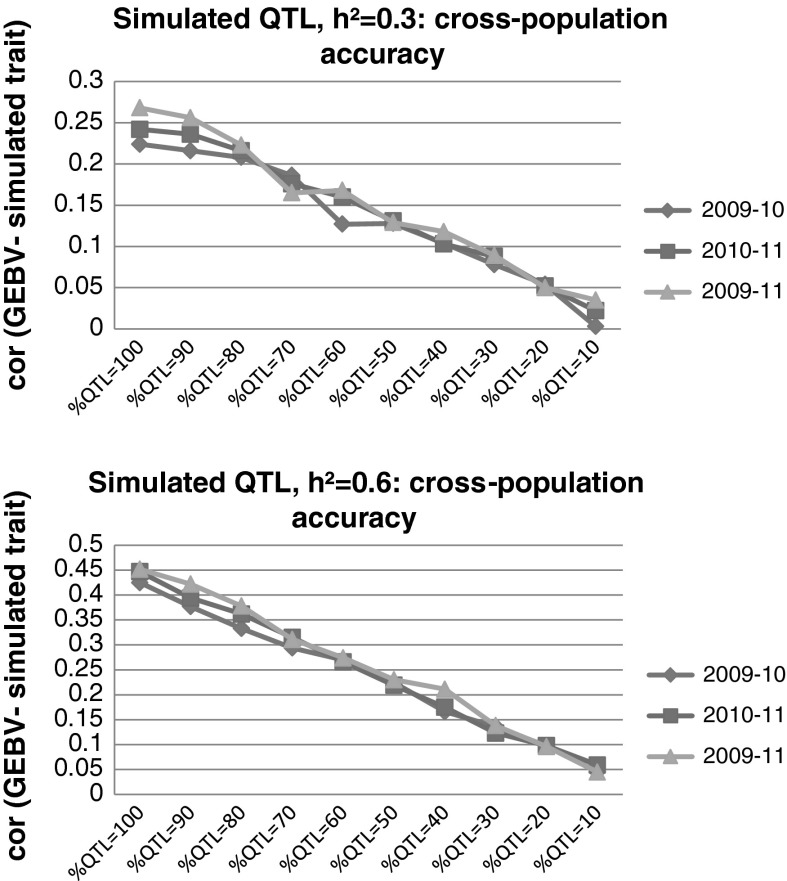
Mean correlations of GEBV and simulated traits in cross-population validation tests, as a function of the percent of QTLs, drawn from a common set of 100 QTLs, in the training and validation populations

For two levels of trait heritability (*h* ^2^ = 0.3 and *h* ^2^ = 0.6), accuracies between predicted and simulated traits decreased as the number of common QTLs between the training and validation sets decreased. For traits with both heritabilities (*h* ^2^ = 0.3 or 0.6), the prediction accuracies were null at QTL < 010 for a proportion of common QTLs of around 10 % (Fig. 3).

### Prediction in the INRA validation set

**Fig. 4:**
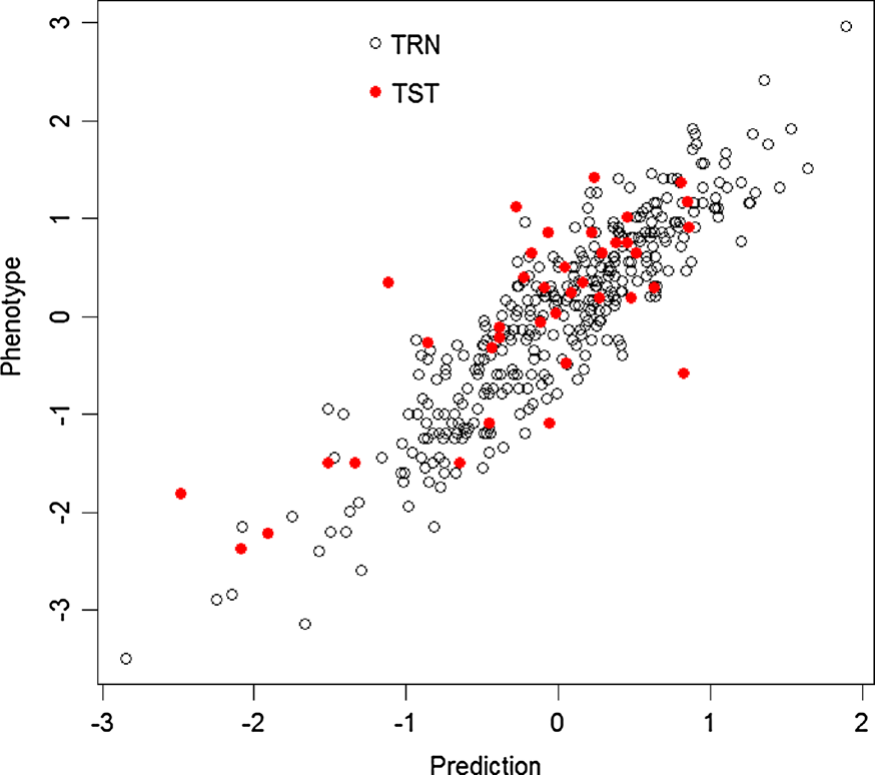
Plot of observed versus predicted value for test weight in the validation set RIL2 (*r* = 0.802)

We used an INRA RIL, population with mean phenotypes over three locations in 2009 for the training set, and a RIL2 population evaluated in 2011 as the validation set. The accuracies estimated with LASSO were *r* = 0.280 for grain yield, *r* = 0.305 for heading date and *r* = 0.802 for test weight (Fig. 4).

### Kinship relationships

The average kinship coefficient between lines within and among populations ranged between 0.329 and 0.42 (Table 1).

**Table 1:** Mean and range of coancestry coefficient among breeding lines within and between populations

	DH1	RIL	DH2	RIL2
DH1	0.42 0.107–0.99	0.341 0.030–0.992	0.387 0.027–0.987	
RIL		0.34 0.034–0.99	0.329 0.0–0.832	0.338 0.035–0.962
DH2			0.381 0.027–0.998	0.335 0.027–0.840
RIL2				0.357 0.106–0.962

## Discussion

The use of standard cross-validation led to highly variable estimates of prediction accuracies between populations and environments. In our investigation, each training population was evaluated for a single year, and the year effect was statistically confounded with the population effect (i.e., genetic background pattern of LD between markers and QTLs). However, three locations were used during each year, which allows us to partly separate the population and environmental effects. Generally, we observed more variation in prediction accuracies between populations within years than between locations within years, particularly for a highly heritable trait, like heading date. This illustrates that the population effect is more important than the location effect, although there were exceptions for the less heritable trait, grain yield, particularly with some statistical methods, like RKHS. Moreover, the ranking of populations for their average accuracies is the same for the three traits, with DH1 (2009) having the lowest and DH2 (2011) having the highest accuracies. This suggests that the population effect, due to genetic architecture (LD and relatedness), is greater than the year effect, which is less likely to be the same for all traits.

Discrepancies in prediction accuracies for the same population evaluated in different environments have been reported for wheat (Crossa et al. 2010; Endelman 2011). For example, Crossa et al. (2010) reported the accuracy for grain yield in four environments ranged from 0.355 to 0.480 using BLUP and from 0.445 to 0.601 using RKHS. Although our accuracies were generally much lower, the best values obtained for grain yield were 0.568 using RKHS and 0.565 using LASSO. The differences in prediction accuracies cannot be accounted for with the training population size because all single populations have similar population sizes (about 350, with 80 % used for training) and there was no improvement when a mixture of two or three populations was included. In theory, the prediction accuracy is positively related to the training population size (Daetwyler et al. 2008, 2010). The lack of relationship found in this study may demonstrate that mixing different breeding populations was not appropriate for building a larger and more efficient training set.

For the same trait/population combination, very similar accuracies were obtained with the different prediction methods, at least for the two traits with high heritabilities. This has already been reported by Heslot et al. (2012), who analyzed three wheat populations for GEBV using ten statistical models, estimating small differences between the populations, with RKHS being the most accurate and support vector machine the least accurate methods. For yield, accuracies ranged from 0.22 to 0.37, which compares to the accuracies estimated in our study. For test weight, Heffner et al. (2011) reported an accuracy of 0.5 using ridge regression on biparental wheat populations. The lower accuracies for yield, compared to other traits, may be due to a lower heritability or to a more complex genetic architecture (i.e., many small QTLs) with information not being totally captured by imperfect marker coverage what about GxE? It seems several studies, including Storlie and Charmet (2013), have suggested G × E is a major confounding factor. Using 41,371 SNP markers from genotyping-by-sequencing 254 advanced breeding lines from CIMMYT, Poland et al. (2012) reported an improvement of 0.1 to 0.2 for yield prediction accuracy over that obtained with 1,726 DArTs. This illustrates that 1,726 DArT markers do not provide sufficient genome coverage to capture all the QTL information. When reducing the SNP markers to the same number as DArT, they still observed an improvement, suggesting that the distribution of DArT markers rather than their number is the main source of lack of accuracy.

Predicting breeding values in a population (grown during a respective year) as a validation set using another population (grown during a respective year) as training set was inaccurate, regardless of the training population size. In this study, there were two possible caveats: There were different populations and different years of evaluation. Heffner et al. (2011) used the phenotype of 1 year for training set and the phenotype of another year as the training set. Accuracies of *r* = 0.199 for grain yield, *r* = 0.560 for test weight and *r* = 0.748 for heading date were estimated (Heffner et al. 2011). For yield, we used separate training and validation sets based on years and showed accuracies were significantly reduced compared to standard cross-validation methods (Storlie and Charmet 2013). These accuracies were higher than the accuracies reported in this study (0.23 vs. 0.00). Therefore, the reduced predictability seems to have causes other than G × E interactions.

Few GS studies have included different populations for training and validation. Lorenz et al. (2012) included one barley population evaluated during 2 years as the training set and another barley population evaluated during a third year as the validation set. Accuracies for Fusarium head blight (FHB) associated traits ranged between *r* = 0.4–0.75 using one population for the training and validation sets, and these accuracies were nearly halved when different populations were used for the sets (Lorenz et al. 2012). Similar results have been reported for similar FHB-related traits in wheat (Rutkoski et al. 2012).

The inaccuracies of cross-population cross-validation in our study may be caused by a lack of genetic relatedness between lines of the training and the validation sets. The coefficient of relatedness between lines of the same breeding population ranged between 0.35 and 0.47 (with 0.50 also measuring full sib families). The two DH populations had higher relatedness levels than the RIL populations, possibly due to fewer parents. The range of relatedness levels did not differ significantly for lines between populations. The relatedness level differed slightly between respective breeding populations (DH1–DH2: 0.387 or RIL1–RIL2: 0.338) versus (DH1–RIL1: 0.341 or DH2–RIL1: 0.329). Relatedness levels may not explain prediction accuracy differences. In another investigation, Zhao et al. (2013) suggested that the prediction accuracy (*r* = 0.28–0.42) was 44 % lower for hybrid wheat when the training and validation sets were not related versus having at least one common parent. One explanation for the reduced accuracies when predicting unrelated populations is the presence of different alleles. Our simulation results suggest a linear relationship between shared alleles and accuracy. Our different populations have a coefficient of relatedness of about 0.3. The relatedness level may correlate with the number of shared alleles and may explain the accuracies of cross-population predictions.

## Conclusion

The elite breeding lines proved to be an interesting support to GS of important traits in wheat. Populations created from lines obtained by several breeders, although of limited size, do present potentially useful accuracy in within-population cross-validation, particularly for the most heritable traits. However, although they were on average similarly related to each other, no gain in accuracy was obtained by mixing one or two breeding populations to make a larger training set. Even more disappointing was the failure of cross-populations validation. This shows that more research is needed and more effort must devoted to design optimal training populations, with a sufficient level of relatedness with the target populations to achieve a good accuracy.

## Electronic supplementary material

Below is the link to the electronic supplementary material.

Supplementary material 1 (DOC 157 kb)
