# Supplementary material for: Genome-wide prediction of three important traits in bread wheat
Source: Mol Breed. 2014 Jul 16;34(4):1843–52. doi: 10.1007/s11032-014-0143-y (PMC4544631; doi:10.1007/s11032-014-0143-y)
Supplement: Supplementary file 1 — Supplementary material 1 (DOC 157 kb) [file 11032_2014_143_MOESM1_ESM.doc]

**Supplementary Material**

**Suppl Table 1:** Summary statistics of Wheat populations DH1, DH2 and RIL: mean, range and heritability of grain yield (t.ha^-1^ 0% humidity), test weight (Kg.hL^-1^) and heading date (days from January 1^st^)

**Suppl Table 2: M**ean correlation (from 200 resamplings) between the observed trait and GEBV from five-fold cross validation within a given population. (Note that GBLUP was discarded, as it did not run on the 2011 population, likely due to excessive relatedness between some lines**.**

**Suppl Table 3: M**ean correlations (from 200 resamplings) between the observed trait (means over the different sites) and GEBV from five-fold cross validations using composite populations, made by combining two or three populations.( Note that GBLUP was discarded, as it did not run on the 2011 population, likely due to excessive relatedness between some lines**.)**

**Suppl Table 4: M**ean correlations (from 200 resamplings) between the observed trait (means over the different sites) and GEBV from cross-population validations (i.e. using sampling 80 of lines in pop1 for training set and 20% of pop2 as validation set**.)**

**Suppl. Table 1**

|  | | | | | |
| --- | --- | --- | --- | --- | --- |
| Population | Trait | Location | Mean | Range | Heritability |
| DH1 | Grain Yield | Capelle | 9.76 | 7.28 – 11.44 | 0.72 |
|  |  | Clermont | 8.86 | 5.85 – 11.08 |  |
|  |  | Milly | 9.62 | 6.49 – 11.27 |  |
|  | Test Weight | Capelle | 78.2 | 72.5 - 83.9 | 0.88 |
|  |  | Clermont | 77.5 | 70.5 - 83.0 |  |
|  |  | Milly | 76.2 | 69.9 - 82.1 |  |
|  | Heading Date | Capelle | 150.6 | 143 - 158 | 0.96 |
|  |  | Clermont | 145.2 | 136 - 153 |  |
|  |  | Milly | 140.5 | 133 - 149 |  |
| DH2 | Grain Yield | Capelle | 10.62 | 7.89 – 12.48 | 0.68 |
|  |  | Milly | 9.75 | 7.39 – 11.84 |  |
|  |  | Rennes | 10.78 | 5.68 – 13.40 |  |
|  | Test Weight | Capelle | 79.4 | 73.4 - 83.2 | 0.76 |
|  |  | Milly | 80.0 | 73.6 - 88.0 |  |
|  |  | Rennes | 75.8 | 68.8 - 81.0 |  |
|  | Heading Date | Capelle | 137.7 | 127 - 151 | 0.95 |
|  |  | Milly | 128.5 | 114 - 140 |  |
|  |  | Rennes | 132.1 | 122 - 141 |  |
| RIL | Grain Yield | Capelle | 10.27 | 6.63 – 13.09 | 0.69 |
|  |  | Clermont | 9.21 | 6.12 – 11.28 |  |
|  |  | Mons | 8.61 | 5.94 – 10.09 |  |
|  | Test Weight | Capelle | 74.3 | 66 - 80.6 | 0.83 |
|  |  | Clermont | 71.2 | 61 - 79.2 |  |
|  |  | Mons | 75.9 | 69.7 - 81.2 |  |
|  | Heading Date | Clermont | 137.7 | 128 - 147 | 0.78 |
|  |  | Mons | 150.4 | 142 - 159 |  |
| DH and RIL: dihaploid and recombinant inbreed lines respectively, from several prebreeding cross | | | | | |

**Suppl.** Table 2

| **Method** | **GBLUP** | **BRR** | **LASSO** | **RKHS** | **Rforest** |
| --- | --- | --- | --- | --- | --- |
| **Yield** |  |  |  |  |  |
| CA09 | 0.172 | 0.185 | 0.195 | 0.126 | 0.166 |
| CF09 | 0.237 | 0.210 | 0.225 | 0.180 | 0.260 |
| MI09 | 0.300 | 0.279 | 0.283 | 0.242 | 0.302 |
| Mean09 | 0.212 | 0.180 | 0.168 | 0.109 | 0.225 |
| CA10 | 0.162 | 0.129 | 0.117 | 0.056 | 0.197 |
| CF10 | 0.315 | 0.337 | 0.354 | 0.331 | 0.376 |
| EM10 | 0.380 | 0.380 | 0.436 | 0.352 | 0.377 |
| Mean10 | 0.266 | 0.278 | 0.216 | 0.231 | 0.305 |
| CA11 |  | 0.548 | 0.565 | 0.568 | 0.570 |
| MI11 |  | 0.534 | 0.518 | 0.522 | 0.572 |
| RE11 |  | 0.241 | 0.231 | 0.194 | 0.345 |
| Mean11 |  | 0.379 | 0.376 | 0.387 | 0.409 |
| **Test weight** |  |  |  |  |  |
| CA09 | 0.240 | 0.267 | 0.259 | 0.255 | 0.279 |
| CF09 | 0.269 | 0.330 | 0.334 | 0.336 | 0.350 |
| MI09 | 0.272 | 0.297 | 0.327 | 0.303 | 0.353 |
| Mean09 | 0.322 | 0.321 | 0.356 | 0.329 | 0.333 |
| CA10 | 0.506 | 0.496 | 0.54 | 0.482 | 0.442 |
| CF10 | 0.417 | 0.420 | 0.509 | 0.415 | 0.462 |
| EM10 | 0.644 | 0.633 | 0.698 | 0.635 | 0.632 |
| Mean10 | 0.594 | 0.595 | 0.702 | 0.595 | 0.583 |
| CA11 |  | 0.653 | 0.648 | 0.656 | 0.670 |
| MI11 |  | 0.650 | 0.638 | 0.646 | 0.635 |
| RE11 |  | 0.653 | 0.647 | 0.657 | 0.670 |
| Mean11 |  | 0.677 | 0.677 | 0.681 | 0.680 |
| **Heading date** |  |  |  |  |  |
| CA09 | 0.493 | 0.484 | 0.471 | 0.489 | 0.478 |
| CF09 | 0.546 | 0.556 | 0.541 | 0.546 | 0.542 |
| I09 | 0.536 | 0.518 | 0.534 | 0.515 | 0.535 |
| Mean09 | 0.538 | 0.548 | 0.544 | 0.552 | 0.527 |
| CF10 | 0.448 | 0.446 | 0.433 | 0.421 | 0.507 |
| EM10 | 0.308 | 0.295 | 0.292 | 0.283 | 0.390 |
| Mean10 | 0.380 | 0.364 | 0.363 | 0.378 | 0.437 |
| CA11 |  | 0.599 | 0.580 | 0.589 | 0.592 |
| MI11 |  | 0.613 | 0.619 | 0.631 | 0.606 |
| RE11 |  | 0.562 | 0.566 | 0.560 | 0.565 |
| Mean11 |  | 0.586 | 0.600 | 0.588 | 0.594 |

| **Suppl. Table 3 :**  **Method** | **BRR** | **LASSO** | **RKHS** | **Rforest** |
| --- | --- | --- | --- | --- |
| **Yield** |  |  |  |  |
| DH1 (n=369) | 0.180 | 0.168 | 0.109 | 0.225 |
| RIL (n=341) | 0.278 | 0.216 | 0.231 | 0.305 |
| DH2 (n=382) | 0.379 | 0.376 | 0.387 | 0.409 |
| DH1+RIL (n=710) | 0.240 | 0.228 | 0.181 | 0.261 |
| DH1+DH2 (n=741) | 0.311 | 0.303 | 0.315 | 0.330 |
| RIL+DH2(n=725) | 0.314 | 0.314 | 0.325 | 0.346 |
| DH1+RIL+DH2(n=1092) | 0.246 | 0.239 | 0.238 | 0.312 |
| **Test weight** |  |  |  |  |
| DH | 0.321 | 0.356 | 0.329 | 0.333 |
| RIL (n=341) | 0.595 | 0.702 | 0.595 | 0.583 |
| DH2 (n=382) | 0.677 | 0.677 | 0.681 | 0.680 |
| DH1+RIL (n=710) | 0.446 | 0.454 | 0.456 | 0.488 |
| DH1+DH2 (n=741) | 0.452 | 0.443 | 0.462 | 0.497 |
| RIL+DH2(n=725) | 0.448 | 0.439 | 0.457 | 0.455 |
| DH1+RIL+DH2(n=1092) | 0.484 | 0.495 | 0.489 | 0.527 |
| **Heading date** |  |  |  |  |
| DH1 (n=369) | 0.548 | 0.544 | 0.552 | 0.527 |
| RIL (n=341) | 0.364 | 0.363 | 0.378 | 0.437 |
| DH2 (n=382) | 0.586 | 0.600 | 0.588 | 0.594 |
| DH1+RIL (n=710) | 0.425 | 0.419 | 0.422 | 0.491 |
| DH1+DH2 (n=741) | 0.597 | 0.602 | 0.604 | 0.584 |
| RIL+DH2(n=725) | 0.553 | 0.562 | 0.569 | 0.612 |
| DH1+RIL+DH2(n=1092) | 0.499 | 0.488 | 0.507 | 0.561 |

| **Suppl Table 4**:   | **Method** | **BRR** | **LASSO** | **Rforest** | | --- | --- | --- | --- | | **Yield** |  |  |  | | RIL using DH1 | -0.078 | -0.080 | 0.015 | | DH2 using DH1 | -0.120 | -0.075 | -0.050 | | DH1 using RIL | -0.044 | -0.049 | -0.018 | | **Test weight** |  |  |  | | RIL using DH1 | 0.225 | 0.243 | 0.050 | | DH2 using DH1 | 0.128 | 0.143 | 0.029 | | DH1 using RIL | 0.090 | 0.085 | 0.059 | | **Heading date** |  |  |  | | RIL using DH1 | -0.058 | -0.062 | -0.017 | | DH2 using DH1 | 0.026 | 0.029 | -0.006 | | DH1 using RIL | 0.058 | 0.081 | 0.031 | |  |  |  |
| --- | --- | --- | --- | --- | --- | --- | --- | --- | --- | --- | --- | --- | --- | --- | --- | --- | --- | --- | --- | --- | --- | --- | --- | --- | --- | --- | --- | --- | --- | --- | --- | --- | --- | --- | --- | --- | --- | --- | --- | --- | --- | --- | --- | --- | --- | --- | --- | --- | --- | --- | --- | --- | --- | --- | --- |
|  |  |  |  |
